# Association of rheumatoid arthritis and its severity with human leukocytic antigen-DRB1 alleles in Kurdish region in North of Iraq

**DOI:** 10.1186/s41927-021-00229-9

**Published:** 2022-01-12

**Authors:** Niaz Albarzinji, Sherzad Ali Ismael, Dashty Albustany

**Affiliations:** 1 Hawler Medical University, Erbil, Iraq; 2 Kurdistan Board of Medical Specialties, Erbil, Iraq

**Keywords:** HLA-DRB1 antigen, Rheumatoid arthritis, Disease susceptibility, Kurdish region, Iraq

## Abstract

**Background:**

Rheumatoid arthritis is a complex multifactorial chronic disease, the importance of human leukocytic antigen (HLA) as a major genetic risk factor for rheumatoid arthritis was studied worldwide. The objective of this study is to identify the association of HLA-DRB1 subtypes with rheumatoid arthritis and its severity in Kurdish region.

**Methods:**

A case–control study recruited 65 rheumatoid arthritis patients and 100 healthy individuals as control group all over the Kurdistan region/Iraq. Both patient and control groups are genotyped using polymerase chain reaction with sequence specific primer. Anti-CCP antibodies were measured by ELISA test. Rheumatoid factor, C-reactive protein, and disease activity score 28 which measured by DAS-28 values were calculated. The DAS-28 was used to assess the clinical severity of the patients.

**Results:**

HLA-DRB1-0404 and HLA-DRB1-0405 frequencies showed a strong association with disease susceptibility (P < 0.001). The frequency of HLA-DRB1-0411 and HLA-DRB1-0413 were significantly higher in control group (P < 0.001). The frequency of rheumatoid factor and Anti-CCP were significantly higher among shared epitope-positive patients compared to shared epitope-negative patients (P < 0.001). Regarding the disease activity by DAS-28, rheumatoid arthritis patients didn’t show significant difference between the shared epitope-positive and shared epitope-negative patients.

**Conclusions:**

HLA-DR0404 and HLA-DR0405 alleles are related to RA, while HLA-DR1-0411 and HLA-DRB1-0413 protect against RA in the Kurdistan region in the North of Iraq.

## Background

Rheumatoid arthritis (RA) is chronic inflammatory disorder of unknown etiology that affect the joints and other organs in the body, regarding the prevalence of the disease is about of 1% [1] of total population. It is characterized by pain and chronic inflammatory arthritis, which leads to progressive destruction of both the bone and the cartilage and leading to functional disability [2].

The disease is more common in women than in men with the ration of 3:1. Although the disease can occur at any age but the peak age of onset of the disease is in the 40s [3]. The genetic and environmental risk factors play key roles in the disease pathogenesis [1, 4]. The inheritance probability of RA is estimated to be around 60% [4, 5].

One of the most important genetic factor that found to be risk factor for developing RA is human leukocytic antigen (HLA),as showed in many studies that HLA-DR4 is associated with developing RA in different populations [6–11]. Recently, nineteen different allelic variants of HLA-DR4 have been found by means of allele specific oligo typing, some of them they are associated with RA in different ethnic groups [6–11]. For example, DRB1*0404 and DRB1*0405 were associated with developing of RA many studies showed that there’s strong relationship between HLA-DRB1 and RA [10, 11]. HLA-DRB1 alleles encoding the SE (DRB1*0401, *0404, *0407, *0409 and *0410) are associated with the severity of RA and have been more recently related with production of anti-citrullinated peptide autoantibodies (anti-CCP) [7–9]. Meanwhile the SE negative genotypes (mainly DRB1*0411 and *0413) are proved to be protective against RA susceptibility [12, 13].

## Methods

The study is a case–control type, recruited 65 patients diagnosed as RA according to American college of rheumatology [14]. The study started in the April 2019 and lasts for 8 months included 18 male and 47 female patients; the cases collected at the rheumatological consultation clinic in Rizgary teaching hospital. Furthermore, the study recruited 100 healthy individuals as control group which are mainly the medical staff. Both patients and control groups were from Kurdish nation in north of Iraq. An informed consent was obtained from all patients and healthy individuals. Both verbal and written informed consents are taken from participants before their participation in the study.

Blood samples were obtained from all the 65 patients and the 100 healthy control cases; the detection of anti-CCP IgG antibodies was performed using ELISA kit (Euroimmun, Lübeck, Germany). Serum samples presenting results > 18 RU/mL were considered to be positive for anti-CCP antibodies. Rheumatoid factor, C-reactive protein (CRP) and disease activity score 28 (DAS-28) values were adopted from patients’ medical records. The DAS-28 was used to assess the clinical severity of the patients [15]. Genomic DNA of patients with RA (n = 65) and healthy controls (n = 100) were isolated from peripheral anticoagulated venous blood samples by using the high pure PCR Template Preparation Kit (Roche, Mannheim, Germany). Genotyping of HLA-DRB1 was performed by polymerase chain reaction with sequence-specific primers (PCR-SSP) using Micro SSPT Generic HLA Class II (DRB) (One Lambda Inc., CA, USA). Odds ratio (OR) and 95% confidence interval (95% CI) were calculated to estimate the strengths of the associations. Chi-squared was used in the statistical analysis. Differences were considered to be significant at P ≤ 0.05. Statistical Package of Social Sciences (SPSS v22) was used for data analysis.

## Results

The mean ± SD of age of the cases was 47.4 ± 10.6 years and the mean ± SD of duration of the disease was 9.5 ± 3.7 years. The mean ± SD of age of the controls was 40.21 ± SD of 10.11 years. All the controls age and gender are matched with the cases. Demographic data and clinical findings of 65 RA patients are diagnosed according to the modified ACR criteria are given in Table 1.

**Table 1:** Demographic and clinical characteristics of patients with rheumatoid arthritis

Characteristics	RA (n = 65)
Age, mean (+ , − SD) years	47.4 (10.6)
Disease duration, mean (+ , − SD) years	9.5 (3.7)
Women	47 (72.30%)
Men	18 (27.69%)
RF positive patients	44 (67.69%)
Anti-CCP positive patients	41 (63.07%)
(RP (mg/L)	25 (32.3)
DAS-28, mean (SD)	5.1 (1.2)

Frequencies of HLA-DRB1 alleles of RA patients and normal individuals with frequency of shared epitope (SE) in RA patients and control group are summarized in Table 2. In RA patients, HLA-DRB1 *0404, *0405 allele frequencies were significantly higher among cases than controls (OR 10.05, 95% CI 4.04—25.02, P = 0.001) and (OR 5.05, 95% CI 2.26–11.27, P < 0.0001) respectively. In contrast, DRB1 *0411 and *0413 alleles were more frequent in controls which was statistically significant (OR 0.15, 95% CI 0.07–0.33, P = 0.001) and (OR 0.13, 95% CI 0.050–0.36), P = 0.001), respectively. The allele frequency differences of DRB1*0403, *0407, *0408, *0409, 0410, *0412, *0414, and *0415 was not statistically significant (95% CI of *16 overlapped). Compared to controls, the frequencies of SE positive alleles (the sum of DRB1*0401, *0404, *0407, *0409 and *0410) were higher significantly in RA patients than the control group (OR 3.41, 95% CI 2.35–4.95, P < 0.0001).

**Table 2:** The distribution of HLA-DRB1 allele frequencies in RA patients and controls

Genotype_HLA_DRB1	RA (n = 65)		Control (n = 100)		OR (95%)	P value
	No	AF (%)	No	AF (%)		
DRB1*0401	5	7.89	6	6	1.306 (0.38–4.46)	0.670
DRB1*0403	8	12.3	21	21	0.52 (0.21–1.276)	0.151
DRB1*0404	28	43.07	7	7	10.05 (4.04–25.02)	< 0.001
DRB1*0405	25	38.46	11	11	5.05 (2.26–11.27)	< 0.001
DRB1*0407	6	9.2	20	20	0.40 (0.15–1.07)	0.063
DRB1*0408	3	4.61	6	6	0.75 (0.18–3.14)	0.702
DRB1*0409	1	1.65	3	3	0.50 (0.051–4.9)	0.969
DRB1*0410	8	12.3	10	10	1.26 (0.47–3.38)	0.642
DRB1*0411	13	20	61	61	0.15 (0.07–0.33)	< 0.001
DRB1*0412	0	0	5	5	0.0 (0.0–?)	0.157
DRB1*0413	5	7.69	38	38	0.13 (0.050–0.36)	< 0.001
DRB1*0414	9	13.84	12	12	1.17 (0.46–2.97)	0.728
DRB1*0415	8	12.3	19	19	0.59 (0.24–1.46)	0.257
SE positive	48	73.84	46	46	3.31 (1.68–6.53)	< 0.001

Shared epitope*0401, *0404, *0407, *0409 and*0410

Values are number (%) unless otherwise indicated

*AF* allele frequency, *SE* positive: the sum of DRB1*0401, *0404, *0407, *0409 and *0410, alleles; *OR* odds ratio; *95% Cl* confidence interval at 95%. HLA frequencies observed in patients and controls were compared using the chi-square test. Differences were considered significant at P < 0.05

Anti-CCP antibody was present in 63.07% while RF was present in 67.69% of the RA patients. Frequencies of anti-CCP antibodies and RF were statistically higher in SE-positive patients compared to SE-negative patients (OR 4.93, 95% CI 1.51—16.08, P < 0.005) and (OR 4.80, 95% CI 1.48–15.59), P < 0.006), respectively, Table 3.

**Table 3:** Association of HLA-DRB1 shared epitopes alleles with anti-CCP and rheumatoid factor antibodies in rheumatoid arthritis patients (n = 65)

SE status	SE positive (n = 48) no. (%)	SE negative (n = 17) no. (%)	OR (95%)	P value
Anti-CCP positive	35 (72.81%)	6 (35.29%)	4.93 (1.51–16.08)	0.005
Anti-CCP negative	13 (27.08%)	11 (64.70%)	0.20 (0.06–0.65)	0.005
RF positive	37 (77.08%)	7 (41.17%)	4.80 (1.48–15.59)	0.006
RF negative	11 (22.91%)	10 (58.82%)	0.20 (0.064–0.67)	0.006

**Fig. 1:**
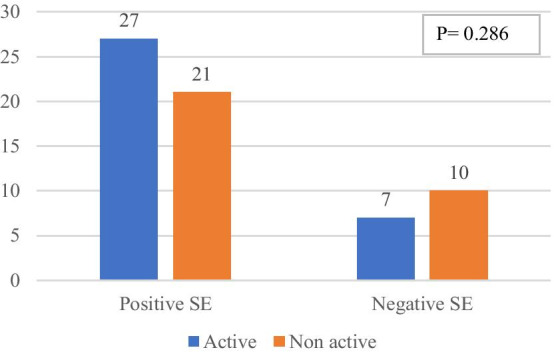
Disease severity by DAS-28

Disease severity presented by DAS-28 values showed no significance difference between SE negative and SE positive RA patients, Fig. 1.

## Discussion

Although the etiology of RA is unclear, but the genetic susceptibility of RA in association with HLA-DR is well known in different ethnic groups such as DR4 in Caucasian, American black, Chinese, and Japanese patients with RA, and DR1 in Asian Indian and Ashkenazi Jewish patients [16–18].

In our study, we have found that the DRB1*0404 and HLA-DRB1*0405 allele to be strongly associated with RA in Kurdish patients. A similar finding was reported in Japanese [10], Singaporean Chinese [11], in Morocco [19] and Zahedan southeast Iran [20], on the other hand, Peruvian [21] and Mexican American [22] populations showed no significant correlation between HLA-DRB1*0404 and RA susceptibility.

The current study showed that HLA-DRB1*0411 and HLA-DRB1*0413 alleles were more frequent in controls than the patients; these alleles were regarded as protective effect against RA. The same result has been reported in several reviews [5, 12, 23, 24] and revealed in different populations. The HLA-DRB1*0411 in Peruvian [25], and Tunisians [26], while HLA-DRB1*0413 showed protective effect against RA in TURKISH [27, 28], Asians [29], and Slovakians [30]. Moreover, HLA-DRB1*0406 showed protective effect in Iranians [20], Saudians [31] and DRB1*0408 in Mexican Americans [2].

The relation between the SEs and the severity of RA has not been clearly understood yet [32] in many studies that done in Northern Europe [33], Netherlands [34], Northern Italy [35], and Caucasians [35, 36]; showed that DRB1*0401 allele is indicated to increase the severity of RA whereas DRB1*0405 allele is mainly in Korea [37]. But in our study showed no significant correlation of disease severity which assessed by mean DAS-28 values between the SE positive and SE negative patients. This may be due to the small number of patients in our study. These results agree with studies carried out in Turkey [28] and Greece [38]. Moreover, our study agree with previously reported relationship of SE positive alleles in the productions of anti-CCP and RF sero-positivity [5, 12, 34, 39].

## Conclusions

HLA-DRB1*0404, and *0405 alleles were proved in our study to be associated with RA while HLA-DRB1*0411 and *0413 were showed to be protective alleles against RA in Kurdish population. No significance was observed between SEs alleles and the severity of RA, both Anti-CCP antibody and rheumatoid factor were significantly higher in SE-positive patients compared to SE-negative patients.

## Data Availability

The datasets used and analyzed during the current study are available from the corresponding author on reasonable request. The blood samples are stored at the center laboratory of Erbil until the end of the study then the specimen containers were discarded into special disinfectant-filled containers.

## Abbreviations

RA: Rheumatoid arthritis
HLA: Human leukocytic antigen
DAS-28: Disease activity score 28
CRP: C-reactive protein
SPSS v22: Statistical Package of Social Sciences version 22
